# Novel Insights on Systemic and Brain Aging, Stroke, Amyotrophic Lateral Sclerosis, and Alzheimer’s Disease

**DOI:** 10.14336/AD.2019.0330

**Published:** 2019-04-01

**Authors:** Ashok K. Shetty, Raghavendra Upadhya, Leelavathi N. Madhu, Maheedhar Kodali

**Affiliations:** Institute for Regenerative Medicine, Department of Molecular and Cellular Medicine, Texas A&M University Health Science Center College of Medicine, College Station, Texas, USA

**Keywords:** aging, amyotrophic lateral sclerosis, Alzheimer’s disease, stroke, brain

## Abstract

The mechanisms that underlie the pathophysiology of aging, amyotrophic lateral sclerosis (ALS), Alzheimer’s disease (AD) and stroke are not fully understood and have been the focus of intense and constant investigation worldwide. Studies that provide insights on aging and age-related disease mechanisms are critical for advancing novel therapies that promote successful aging and prevent or cure multiple age-related diseases. The April 2019 issue of the journal, "Aging & Disease" published a series of articles that confer fresh insights on numerous age-related conditions and diseases. The age-related topics include the detrimental effect of overweight on energy metabolism and muscle integrity, senoinflammation as the cause of neuroinflammation, the link between systemic C-reactive protein and brain white matter loss, the role of miR-34a in promoting healthy heart and brain, the potential of sirtuin 3 for reducing cardiac and pulmonary fibrosis, and the promise of statin therapy for ameliorating asymptomatic intracranial atherosclerotic stenosis. Additional aging-related articles highlighted the involvement of miR-181b-5p and high mobility group box-1 in hypertension, Yes-associated protein in cataract formation, multiple miRs and long noncoding RNAs in coronary artery disease development, the role of higher meat consumption on sleep problems, and the link between glycated hemoglobin and depression. The topics related to ALS suggested that individuals with higher education and living in a rural environment have a higher risk for developing ALS, and collagen XIX alpha 1 is a prognostic biomarker of ALS. The topics discussed on AD implied that extracellular amyloid β42 is likely the cause of intraneuronal neurofibrillary tangle accumulation in familial AD and traditional oriental concoctions may be useful for slowing down the progression of AD. The article on stroke suggested that inhibition of the complement system is likely helpful in promoting brain repair after ischemic stroke. The significance of the above findings for understanding the pathogenesis in aging, ALS, AD, and stroke, slowing down the progression of aging, ALS and AD, and promoting brain repair after stroke are discussed.

The population of older adults is increasing greatly in virtually every country in the world because of the rise in life expectancy. As per data from the United Nations (World Population Prospects: the 2017 Revision), the number of older individuals (i.e., those aged 60 years or over) in the world is projected to enlarge from 962 million in 2017 to 2.1 billion in 2050. However, increased life expectancy does not necessarily translate into successful aging for all aged individuals. Successful aging is a broad term referred to individuals in the 60-80 age group exhibiting no significant disease or disability, sustaining a reasonable cognitive function and participating actively with life. Unfortunately, aging is the predominant risk factor for most diseases and conditions that limit healthspan [1]. Indeed, a significant percentage of older individuals develop one or more age-related diseases [2], which may include atherosclerosis [3], hypertension [4], cancer [5], type 2 diabetes [6], cataract [7], age-related macular degeneration [8], Parkinson’s disease [9], Amyotrophic lateral sclerosis (ALS) [10], stroke, [11] dementia [12] and Alzheimer’s disease (AD) [13]. Thus, to enhance the overall quality of life, comprehending the mechanisms of age-related diseases is critical, which may help in developing apt intervention therapies that decelerate the pace of aging as well as diminish or defer the prevalence of age-related diseases [1, 14, 15].

The April 2019 issue of the journal, "Aging & Disease" comprises articles that provide fresh insights on multiple age-related conditions and diseases. The discussed topics related to aging include the effect of overweight on energy metabolism and muscle integrity [16], chronic inflammation in aging and age-related diseases [17], links between C-reactive protein levels and corpus callosum changes [18], microRNAs and genetic nexus of brain aging [19], cardiac and pulmonary fibrosis [20], asymptomatic intracranial atherosclerosis [21], involvement of mir-181b-5p and high mobility group box-1 (HMGB1) pathway in hypertension [22], the role of Yes-Associated protein in cataract formation [23], the role of miRNAs in diagnosis and prognosis of coronary artery disease [24], meat consumption and sleep [25], and links between glycated hemoglobin and depression [26]. Furthermore, two articles discussed the influence of environment and lifestyle on the incidence and progression of ALS [27] and the efficacy of collagen XIX alpha 1 for improving the prognosis of ALS [28]. Besides, another set of articles confer processing of mutant beta-amyloid precursor protein in familial AD [29], the promise of traditional oriental medicines for slowing down the progression of AD [30], and the significance of complement system in stroke [31]. This commentary confers the highlights and the relevance of some the above issues to promote successful aging, to slow down the progression of ALS and AD, and promote brain repair after stroke.

## Overweight alters energy metabolism and muscle integrity in aged individuals

Functional decline in the elderly is associated with sarcopenia, which is a progressive age-related loss of skeletal muscle mass with ~50% reduction by 80 years of age [32]. Sarcopenia has various implications, which include alterations in neuromuscular junction, hormone production, bone mineral density weakness, and loss of stamina. It is well known that obesity aggravates the age-related loss of skeletal muscle mass and contributes to frailty. However, the mechanisms triggering these processes are unclear. Potes and colleagues report that, in elderly male and female individuals, overweight leads to loss of skeletal muscle mass through a switch in energy metabolism [16]. They demonstrated that muscle in overweight, aged individuals exhibited reduced Adenosine-5′- triphosphate (ATP) production, increased mitochondrial genesis coupled with reduced mitophagy resulting in dysfunctional mitochondria. A reduced mitophagy occurred due to reduced levels of mitochondrial fusion proteins mitofusin 2 and optin atrophy protein 1. As a consequence of these changes, a metabolic switch occurred from oxidative to lactic acid fermentation metabolism, which allowed continued ATP production under mitochondrial dysfunction but did not reach physiological levels seen in aged individuals with healthy weight [16]. The effects of ATP depletion were apparent from early signs of impaired contractile function and decreased skeletal muscle structural integrity. Overall, this study provided a novel insight into the main effector pathway at an early stage of obesity and the significance of mitochondrial metabolism in overweight individuals. Thus, approaches that normalize mitochondrial function may slow down the loss of muscle mass in older adults with overweight or afflicted with other age-related diseases.

## Senoinflammation causes chronic inflammation in aging and age-related diseases

The inflammatory process, an immunological defense system in living organisms, is necessary for the survival of species. An acute inflammatory reaction lasting for a short duration is beneficial as such response can eliminate pathogens, toxins, or allergens. A tightly coordinated activity of immune cells, endogenous anti-inflammatory proteins, and tissue remodeling processes facilitate the resolution of acute inflammation and restore homeostasis [17, 33]. When an acute inflammatory response fails to resolve, chronic inflammation ensues with the mobilization of more defense components to create a long-term unresolved immune response [34]. Chronic inflammation is typically linked with changes in the cellular redox state and cell death signaling pathways [17, 35].

With advanced age, dysregulation of the immune response leads to a chronic systemic inflammatory state. It is recognized that cytokines and chemokines are significant culprits in the development of chronic inflammation and the immunosenescence process. Age-associated chronic inflammation can exacerbate the aging process and age-related chronic diseases. Chung and associates discuss newly emerging data on multi-phase inflammatory networks and proinflammatory pathways relevant to aging [17]. They point out that aging is associated with enhanced nuclear factor kappa beta (NF-κB) signaling, and elevated levels of cytokines, chemokines, endoplasmic reticulum stress, inflammasome, and lipid accumulation. Importantly, they propose that senoinflammation is the cause of age-related inflammation, which contradicts earlier hypotheses that inflammaging and molecular inflammation underlie age-related inflammation. Inflammaging is a chronic low-grade inflammation resulting from the response of innate immune cells to misplaced and misfolded molecules from damaged cells [36]. Whereas, molecular inflammation is typified by chronically elevated levels of pro-inflammatory mediators such as tumor necrosis factor alpha (TNFa), interleukin-1 beta (IL-1b), interleukin-6 (IL-6), cyclooxygenase-2 (COX-2), and inducible nitric-oxide synthase (iNOS) during the aging process as a result of an age-related redox imbalance activating many proinflammatory signaling pathways including the transcriptional factor NF-κB [37]. The concept of senoinflammation, on the other hand, is based on three separate phases that are functionally interdigitated, ranging from the redox-sensitive core transcription factor NF-κB and polarized macrophages to miRNAs and metabolically linked proinflammatory processes such as endoplasmic reticulum stress and autophagy [17]. The authors propose that the senoinflammation concept offers molecular insights on complex interactions occurring between distinct transcription factors, inflammatory mediators, and proinflammatory metabolic pathways as being integral. Overall, this new idea has merit and provides a broader scope to examine an intricate network of many inflammatory mediators causing chronic systemic inflammation.

## Controlling systemic C-reactive protein levels may preserve white matter integrity in the brain

To understand the role of chronic low-grade inflammation on the integrity of brain white matter, Cyprien and colleagues examined whether, in older adults without dementia, higher serum levels of high-sensitivity C-reactive protein (hs-CRP) are associated with reduced size of the corpus callosum [18]. Corpus callosum is the largest white matter region in the brain comprising commissural fibers connecting the two cerebral hemispheres [38]. In this study, hs-CRP testing and structural magnetic resonance imaging were conducted on French community-dwelling subjects aged 65 and older. The study found that anterior, mid, and total midsagittal regions of the corpus callosum were smaller with higher hs-CRP level. Authors point out that the associations between hs-CRP level and smaller anterior and total midsagittal corpus callosum areas were significant even after adjustment for body mass index, diabetes, inflammation-related chronic pathologies, and white matter lesions. While correlative, the results suggested that low-grade inflammation in older adults is linked with the altered structural integrity of the corpus callosum [18]. However, it was unclear whether the smaller corpus callosum was a result of degeneration of commissural axons or demyelination due to dysfunction of oligodendrocytes or their precursors. Nonetheless, the results have significance as corpus callosum abnormalities are apparent in neurodegenerative diseases [39], mood disorders [40] and individuals with suicidal behavior [41]. Also, the corpus callosum is one of the first regions to show signs of aging-related degeneration [42]. However, it remains to be seen whether normalization of hs-CRP levels through lifestyle changes would reverse adverse alterations in the corpus callosum of older adults.

## MicroRNA-34a may be a target for promoting healthy heart and brain during aging

An intricate, cohesive and progressive deterioration of cellular activity in specific organs of the body is a feature of aging, which increases the risk for developing diseases such as cancer, diabetes, cardiovascular disorders, stroke, dementia and AD [19]. Sarkar and associates discuss in detail the genes and their functional pathways implicated in brain aging to develop approaches targeted to block or slow down the neuropathological processes [19]. They highlight how specific microRNAs (miRs) can alter the expression of genes involved in neuroinflammation, acute neuronal injury and chronic neurodegenerative diseases such as AD, Parkinson’s disease and stroke. Notably, they suggest that anxiety in AD is likely linked to an elevated expression of miR-92a in the AD brain. The suggestion is based on findings that miR-92a inhibits the synthesis of vesicular gamma-aminobutyric acid (GABA) transporter (VGAT), which is a protein involved in loading the inhibitory neurotransmitter GABA into secretory vesicles [43]. This alteration leads to decreased GABA signaling and anxiety. Likewise, downregulation of miR-132 and miR-212 in AD brain enhances the expression of nitric oxide synthase 1, which activates a deleterious cascade that results in tau hyperphosphorylation and neurodegeneration [44]. Importantly, the authors point out that miR-34a is a good candidate as a biomarker for the aging heart and brain. The proposition is based on increased expression of miR-34a in aged mice and humans as well as the role of miR-34a in inducing telomere attrition, DNA damage responses, apoptosis of cardiomyocytes, and deterioration of recovery after acute myocardial infarction in the aging heart. Furthermore, in aging and AD brains, miR-34a targets genes related to neural activity-dependent grey and white matter plasticity, angiogenesis, neurodegeneration, and memory dysfunction [19]. Since miR-34a promoter contains binding sites for transcription factors such as p53, NF-kB, and signal transducer and activator of transcription 3 (STAT3) [45], they hypothesize that activation of these transcription factors in aging tissues as a result of enhanced reactive oxygen species and inflammaging leads to increased miR-34a expression. Thus, blocking the expression of miR-34a via anti-miR drugs might promote successful aging of the heart and brain.

## Sirtuin 3 (SIRT3) is a promising candidate for reducing cardiac and pulmonary fibrosis associated with aging

Aging raises the probability for developing cardiac and pulmonary fibrosis, as a result of increased frequency of heart failure and fibrotic respiratory diseases in old age [20, 46, 47]. Fibrosis, exemplified predominantly by the accretion of extracellular matrix component collagen at the site of injury, is an essential aspect of wound healing and tissue repair. Unremitting accumulation of collagen is damaging and a common pathological activity in cardiovascular and respiratory diseases [20, 48]. Murtha and colleagues conferred the role of cellular senescence, inflammaging, autophagy and mitochondrial dysfunction in fibrosis [20]. They suggest that age-related alterations such as cellular senescence and inflammaging could reduce the regenerative potential of damaged cardiac and pulmonary tissues, which in turn could increase the likelihood for developing pathological fibrosis after injury. They add that cellular senescence and inflammaging are considered beneficial at lower levels because cellular senescence can protect the organism against cancer and inflammaging can regulate immune response to get rid of invading pathogens and cellular debris. However, increased levels of senescence and inflammaging observed in older individuals can promote cardiac fibrosis and idiopathic pulmonary fibrosis. The authors suggest that strategies that promote autophagy and/or mitochondrial turnover with the regulation of mitochondrial fission and fusion may prevent fibrosis [20]. Notably, they propose that enhanced expression of SIRT3, a mitochondrial protein deacetylase and regulator of antioxidant response and mitochondrial homeostasis, may reduce cardiac and pulmonary fibrosis in aging. The suggestion is based on observations of reduced SIRT3 expression in the lungs of old mice and two murine models of fibrosis, and spontaneous arterial hypertension and cardiac fibrosis observed in mice lacking SIRT3 [49-51]. However, studies are needed in the future to determine whether activation of SIRT3 would be sufficient to prevent cardiac and pulmonary fibrosis associated with aging.

## Long-term statin therapy ameliorates asymptomatic intracranial atherosclerotic stenosis

Symptomatic intracranial atherosclerotic stenosis is a common cause of ischemic stroke worldwide and a high-risk factor for subsequent stroke recurrence [21] whereas, the risk of stroke from asymptomatic intracranial atherosclerotic stenosis (AICAS) is considered relatively low (~3%). However, the prevalence of AICAS could increase to ~30% for patients exhibiting vascular risk factors such as advanced age, high blood pressure, high cholesterol, and diabetes [52]. Besides, mild to moderate AICAS is an independent risk factor for future ischemic stroke in a healthy population [21]. Considering these, treating AICAS has considerable significance. Miao and associates examined whether intensive lipid-lowering therapy using statin would ameliorate asymptomatic intracranial atherosclerosis [21]. They enrolled seventy-one AICAS patients (30-80 years Chinese patients with hyperlipidemia) and evaluated vascular stenoses with transcranial color-coded sonography both before and after statin treatment. Follow-up was done for two years in two cohorts of patients, one with intensive statin treatment and another with standard statin treatment. They found that the degree of stenosis in AICAS patients can be reduced with intensive lipid-lowering therapy within two years. Also, the target LDL-C level was reached by moderate-intensity statin treatment for AICAS patients. However, the study did not seem to analyze the efficacy of statin therapy for reducing vascular stenoses in young AICAS patients vis-à-vis aged AICAS patients.

## MicroRNA-181b-5p and HMGB1 are involved in hypertension

Hypertension is a wide-spread public-health challenge with over a third of people worldwide exhibiting varying levels of hypertension [53]. Several mechanisms likely play a role in the progression of hypertension, which may include excessive vasoconstriction, deficient vasodilatation, and phenotypic transformation of vascular smooth muscle cells (VSMCs) [54]. Because high mobility group box-1 (HMGB1) protein has been reported to be involved in several pathogenic processes including VSMC proliferation and migration, Li and co-workers examined the role of HMGB1 in VSMC phenotypic transformation in hypertension [22]. Their study showed elevated HMGB1 in a cell culture model of angiotensin II-induced VSMC phenotypic transformation associated with down-regulation of contractile proteins and up-regulation of synthetic proteins [22]. Besides, the phenotypic alteration could be blocked through knockdown of HMGB1 or treatment with an angiotensin II receptor blocker losartan. They also found that miR-181b-5p was significantly down-regulated in angiotensin II-treated cells and treating cells with miR-181b-5p mimicked diminished HMGB1 expression and the phenotypic transformation of VSMCs [22]. Furthermore, analyses of plasma from hypertensive male and female patients in the age range of 18-78 years revealed a decreased concentration of miR-181b-5p with increased levels of angiotensin II and HMGB1, but the administration of angiotensin receptor blockers reversed these effects. From these results, authors suggest that down-regulation of miR-181b-5p causes elevation of HMGB1 in hypertensive patients, which in turn promotes a phenotypic transformation of VSMCs and vascular remodeling. The study also indicated that miR-181b-5p and HMGB1 together could serve as biomarkers for vascular remodeling in hypertensive patients.

## Yes-Associated protein deficiency promotes cataract formation

A cataract, typified by clouding of the usually clear lens of the eye, is the most common disease that causes blindness. The risk factors for developing cataract include increasing age, diabetes, excessive exposure to light, smoking, obesity, hypertension, and previous eye injury or surgery. While the precise mechanisms are unclear, a combination of factors has been suggested to play roles in cataract formation [23]. These include dysregulated proliferation and apoptosis of lens epithelial cells, abnormal lens fiber cell differentiation and denucleation, cellular senescence, oxidative stress and inflammation [23, 55-57]. A study by He and colleagues examined the role of Yes-associated protein (Yap), a downstream effector of the Hippo signaling pathway in cataract formation. The authors report that specific expression of Yap is seen in lens epithelial cells and conditional knockout of Yap leads to cataract formation. The lens deficient in Yap demonstrated a smaller number of epithelial cells, preservation of nuclei and accretion of morgagnian globules in the transitional and posterior areas. Furthermore, studies in Yap conditional knockdown mice demonstrated reduced proliferation of epithelial cells, delayed fiber cell denucleation and increased cellular senescence in the lens. Also, RNA profiling analysis revealed that Yap knockdown results in considerable changes in gene transcription that are related to the development of the eye, the structure of lens, inflammation, and the proliferation of cells. Thus, Yap deficiency appears to be involved in cataract formation, and modulation of Yap expression in the lens may prevent or slow down cataract formation.

## Multiple microRNAs and long noncoding RNAs are involved in coronary artery disease development

Cardiovascular disease, particularly coronary artery disease (CAD), is the foremost cause of mortality worldwide [24]. The buildup of atherosclerotic plaques in the wall of the coronary arteries leads to CAD [24, 58]. The pathogenesis of CAD results from alterations in multiple cell types in the artery walls, which comprise endothelial cell (EC) dysfunction, vascular smooth muscle cell (VSMC) alteration, lipid deposition and macrophage activation [24]. Zhang and colleagues reviewed the literature on molecular mechanisms by which miRNAs and long noncoding RNAs (lncRNAs) influence CAD [24]. They report that several miRNAs and lncRNAs are critical regulators in processes related to CAD caused by atherosclerotic lesions. Many miRNAs and lncRNAs play crucial roles in several aspects of these processes, which include lipid metabolism, endothelial cell dysfunction, VSMC phenotype, cholesterol transport, foam cell formation, and vascular inflammation. For example, CAD patients exhibit reduced expression of miR-126-5p [24, 59] and diminished expression of miR-126-5p expression is involved in plaque formation and augmented leukocyte adherence to endothelial cells via endothelial vascular cell adhesion molecule-1 [24, 60]. Likewise, the miR-17-92 cluster, which exhibits downregulation in patients with CAD, is closely linked with TNFa-induced apoptosis of endothelial cells [24, 61]. The authors also suggest that several miRNAs and lncRNAs have potential as biomarkers and therapeutic targets for the CAD. However, additional detailed studies on the function of miRNAs and lncRNAs in CAD will be critical to elucidate mechanisms by which they promote or block CAD.

## Higher meat consumption cuts sleep duration

Sleep problems can lead to adverse health outcomes in older people. A significant alteration in sleep duration and/or quality are linked to cardiometabolic diseases, cognitive decline, and frailty [25, 62-67], implying that sleep disorder can aggravate the outcomes of the aging process. Hence, maintenance of physical and mental function in old age also depends on achieving a good sleep pattern. Serotonin and melatonin represent two neurotransmitters important for biological rhythms such as sleep and alertness. Since amino acids required for the synthesis of these neurotransmitters come from diet, Lana and colleagues examined the association of chronic meat consumption with changes in sleep duration and with sleep quality in older adults [25], using three years of data from 1,341 participants aged ≥60 years. The authors report that in comparison to individuals in the lowest meat consumption (<87 g/d), those in the highest meat intake (≥128 g/d) exhibited a substantial decline (≥2 h) in sleep duration. Greater intake of meat was also allied with the prevalence of snoring and poor general sleep quality. The overall results were similar across individuals who consumed red and processed meat or white meat. Besides, a greater level of meat intake (≥128 g/d) was associated with poor sleep in older adults. The authors suggest that, since high protein intake is required for the older population to prevent frailty and sarcopenia, it will be essential to identify types of food that not only provides high-quality protein and other nutrients as well as promotes positive effects on sleep patterns.

## Increased levels of glycated hemoglobin are associated with depression in old age

Approximately 15% of the population is afflicted with depression, and a major depressive disorder is associated with considerable morbidity and disability [68]. Li and coworkers assessed links between diverse trajectories of depressive symptoms and glycated hemoglobin (HbA_1c_) concentration [26]. The rationale for correlating HbA_1c_ level with depressive symptoms stems from results of previous studies demonstrating an association between HbA_1c_ and depressive symptoms [26, 69, 70]. The investigators employed 10-item Center for Epidemiological Studies-Depression scale in three visits performed in 2011, 2013 and 2015 among 9,804 participants with a mean age of 60.0 ± 9.0 years. HbA_1c_ was measured at baseline, and the participants were categorized into five groups as per the respective quintile. Four distinct trajectories of depressive symptoms were identified, which comprised low symptoms, decreasing symptoms, increasing symptoms, and high symptoms. The authors report that after adjusting for demographic, health-related, and cognitive factors, the risk ratio with the highest HbA_1c_ (Quintile 5) for decreasing, increasing, and high symptoms of depression versus low symptoms was 1.01, 1.12, and 1.39 compared with the lowest HbA_1c_ (Quintile 1), respectively. The study revealed that high levels of glycated hemoglobin concentrations were associated with a much higher risk for developing increasing and high-stable symptoms of depression. The mechanisms underlying the link between HbA_1c_ levels and high symptoms of depression are unclear, however. Authors speculate that both vasculature and functional areas in the brain are likely more vulnerable to poorer glycemic control [26, 71]. Additional brain imaging studies will be useful in the future to know whether the size of the hippocampus correlates with the HbA_1c_ concentration, as reduced size of the hippocampus, likely due to waned neurogenesis, is one of the hallmarks of major depressive disorder [72, 73].

## Individuals with higher education and living in a rural environment have a higher risk for developing ALS

A progressive dysfunction or loss of upper and/or lower motor neurons in the brain and spinal cord typify the neurodegenerative disorder ALS, which eventually leads to paralysis of voluntary muscles, with a variable proportion of spasticity and atrophy [74]. Most ALS cases are sporadic, but 5-10% of the cases are familial ALS. While all types of ALS are equally incurable, considerable variability exists within ALS, with the heterogeneity of initial presentation, the progression of the disease, and survival [75, 76]. Significant clinical heterogeneity in the onset and progression of ALS may be related to lifestyle and environmental factors. To examine such links, Korner and colleagues analyzed a cohort of 117 German ALS patients and 93 controls for physical activity, dietary habits, smoking, residential environment, potentially toxic environmental factors and profession before symptom onset and throughout the disease course [27]. ALS patients and controls did not differ in terms of smoking, diet, the extent of physical training or frequency of toxic influences in this study. However, the ALS patients lived in the rural environment more often than the control persons, but ALS patients did not have a higher percentage of occupation in agriculture [27]. Interestingly, the ALS group had a higher percentage of university graduates. Analyses showed that patients with bulbar onset of ALS, typically associated with cognitive alterations [77], were more often born in an urban environment, in comparison to patients with spinal onset of ALS [27]. Apart from education and environment, ALS phenotypes did not differ in any investigated environmental or lifestyle factor [27]. From these data, authors conclude that no correlation exists between the onset or the rate of ALS progression and any of the measured lifestyle and environmental factors. A surprising finding in this study is that individuals having higher education and living in a rural environment seem to have a higher risk of developing ALS. As suggested by authors, larger multicenter studies are necessary to validate these results.

## Collagen XIX alpha 1 is likely a prognostic biomarker of amyotrophic lateral sclerosis

There is a need for reliable diagnostic or prognostic biomarkers in age-related neurodegenerative diseases such as ALS. Cross-sectional studies indicate that neurofilament protein levels may provide prognostic information for survival in patients with mutations in the gene C9orf7 [78]. However, reliable prognostic biomarkers of ALS mirroring neurodegeneration are yet to be discovered. Calvo and associates performed a study in 268 participants from three cohorts to identify a reliable prognostic biomarker of ALS [28]. The muscle and blood cohorts were investigated in two cross-sectional studies, while the serial blood cohort was evaluated in a longitudinal study at 6-monthly intervals. Fifteen target genes and fourteen proteins involved in muscle physiology and differentiation, metabolic processes and neuromuscular junction dismantlement were examined in the three cohorts. In the muscle biopsy cohort, the risk for higher mortality in an ALS patient showing high Collagen type XIX, alpha 1 (COL19A1) protein levels and a fast progression of the disease was ~71%, while in the blood cohort, this risk was 20%. In the serial blood cohort, there was a significant association between increasing *COL19A1* gene levels and faster progression of the disease during the follow-up period of 24 months [28]. Besides, higher *COL19A1* levels and quicker progression increased the mortality risk. From these results, authors propose that *COL19A1* could be a useful prognostic biomarker for selecting a homogeneous group of patients for clinical trials and suggest that *COL19A1* is likely a promising therapeutic target in ALS [28].

## Extracellular Aβ-42 causes the accumulation of intraneuronal NFTs in familial AD

Nearly 47 million people have dementia in the world, and this population is projected to reach ~132 million in 2050. AD, an age-related neurodegenerative disease typified by a progressive loss of memory, is the most common cause of dementia. Individuals with AD present difficulties in communication, learning, judgment, reasoning, apathy, eating and sleeping disorders, and depression [79, 80]. While the precise causes of AD are still unknown, it is generally deemed that AD occurs as a result of multiple factors, which include genetics (e.g., presence of a risk gene apolipoprotein allele E4 (APOE4) and lifestyle and environmental factors such as heart disease, stroke, high blood pressure, diabetes, and obesity [81]. Deposition of amyloid plaques in the extracellular space and neurofibrillary tangles within neurons are the two conspicuous alterations in the brain that are believed to contribute to the progression of AD symptoms. Amyloid plaques are aggregates of a protein called amyloid-beta (A-beta), which are released from the amyloid precursor protein (APP) through sequential cleavage by beta and gamma secretases [80]. On the other hand, neurofibrillary tangles are aggregates of another protein called tau [81]. With an increasing accumulation of plaques and tangles, additional changes such as neuroinflammation, impaired transfer of information at synapses, loss of synapses, and neurodegeneration occur [82].

While it is still controversial whether plaques are the cause or the result of AD pathogenesis, some studies imply that single pathogenic mutations in APP or presenilin 1 or 2 can cause AD with most of the clinical and neuropathological features [29]. Bi and associates performed a comprehensive review of the literature, comprising clinical, neuropathological, cellular and animal model data from sources such as PubMed and multiple AD databases. Pearson correlation analysis combining the clinical and neuropathological data and aspects of mutant APP processing in cellular models was performed. Their findings suggest that increased Aβ42 correlates with the appearance of neurofibrillary tangles (NFTs) and an earlier age of AD onset. On the other hand, increased Aβ40 is associated with enhanced age at death. Additional observation of interest is a negative correlation between increased α-carboxyl terminal fragment (CTF) and the age of AD onset. Moreover, animal model investigations suggested intracellular Aβ as a critical component for developing memory impairments. Based on these results, authors propose that extracellular Aβ42 causes the accumulation of intraneuronal NFTs, and increased intraneuronal APP proteolytic products (CTFs and Aβs) induce cellular organelle stress that leads to neurodegeneration in AD [29]. The proposed hypothesis has merit for understanding the process of neurodegeneration after the onset of AD (i.e., after the accumulation of Aβ). However, insights on the causes of Aβ42 accumulation and early pathological changes are also crucial for treating AD, as therapeutic attempts focused solely on Aβ accumulation have failed [83].

## Role of microglia and astrocytes in the pathogenesis of AD

Increasing evidence now suggests a role for neuroinflammation in the early pathogenesis of AD. Neuroinflammation, particularly the activation of microglia, in the early stage of AD is considered beneficial for Aβ clearance. However, prolonged activation of microglia can cause damaging effects through altered phagocytosis impeding the removal of Aβ, aberrant synaptic pruning promoting synaptic loss and the release of proinflammatory cytokines changing the milieu as well as astrocyte function [84]. Astrocytes likely transform into toxic type 1 astrocytes in such microenvironment. In normal conditions, astrocytes maintain homeostasis of the brain by providing trophic and metabolic support to neurons, recycling neurotransmitters, stimulating synaptogenesis and synaptic neurotransmission, supporting the blood-brain barrier, and regulating regional blood flow [85]. When instigated by microglia, astrocytes become reactive and play significant roles in the neuroinflammatory and neurodegenerative processes in AD. Indeed, the findings in a mouse study have demonstrated that astrocytes are promoters of neurodegeneration in AD after instigation by microglia [85, 86]. Activated microglia secrete interleukin-1 alpha (IL-1a), TNFa, and complement component 1q (C1q), which together induce the A1 neurotoxic phenotype [86]. Mouse A1 reactive astrocytes upregulate expression of genes of the complement cascade, including complement component 3 (C3) and release an unidentified neurotoxin that induces the death of neurons and oligodendrocytes [87]. Moreover, mouse A1 astrocytes show decreased ability to promote synapse formation and function, to phagocytose synapses and myelin debris, and to promote neuronal survival and growth [86]. About 60% of the astrocytes in the prefrontal cortex of post-mortem brains of patients with AD are expressing C3 [85, 86], which may be human A1 neurotoxic astrocytes, The A1 neurotoxic phenotype, therefore, might represent part of a generic pathway in neurodegeneration. Thus, therapeutic strategies that modulate activated microglia and reactive astrocytes in the early stage of AD may be beneficial for blocking or at least restraining the progression of pathological changes and cognitive impairments.

## Traditional oriental medicines may be useful for slowing down the progression of AD

There is no cure for AD currently. The approved drugs are mostly symptom-relieving drugs, which include acetylcholinesterase inhibitors donepezil, galantamine and rivastigmine, and N-methyl D-aspartate receptor blocker memantine. Also, therapeutic attempts focused solely on removing Aβ plaques have failed to improve cognitive function in AD [83]. Thus, disease-modifying drugs for AD are urgently needed. The development of multi-target drugs that ameliorate different symptoms of AD may be more beneficial for slowing down the disease process [30]. Jeon and colleagues reviewed the efficacy of traditional oriental medicines (TOMs) for AD [30], as some recent studies have suggested potent therapeutic effects of pharmacological compounds found in TOMs for treating AD [88-90]. Since the concoction of traditional medicines from East Asian countries, including China, Korea, and Japan, commonly consist of multiple herbs, the authors reviewed the effects of mixtures of standardized formulae of TOMs [30]. The authors report that one of the most notable impacts of the formulae is inhibition of Aβ accumulation. They particularly highlight that a treatment regimen comprising a herbal formula named GRAPE (encompassing *Panax ginseng*, *Rehmannia glutinosa*, *Acorus tatarinowii*, *Polygala tenuifolia*, *Epimedium brevicornu, Cornus officinalis, Cistanche deserticola, Curcuma aromatica, Salvia miltiorrhiza, Angelica sinensis, Gastrodia elata, Coptis chinensis*) and AD approved drugs donepezil and/or memantine was more beneficial on cognitive function than treatment with conventional drugs alone [91]. Additional studies showed that *Bushen Tiansui* decoction (comprising *Epimedium brevicornum Maxim*, *Polygonum multiflorum Thunb*, *Chinemys reevesii, Fossilia Ossis Mastodi, Polygala, Acorus tatarinowii*) and triterpenoid saponins of *Xanthoceras sorbifolia* Bunge decreased hippocampal damage and cognitive deficits induced by Aβ aggregation [92, 93]. Based on these results the authors recommend clinical trials provided more rigorous studies are conducted first on toxicity, pharmacokinetics, and pharmacodynamics. These are important issues, as some TOMs may contain metals, pesticides, and microorganisms. Thus, TOMs may have promise for treating AD, but validation will require rigorous additional long-term studies in animal models as well as double-blind, placebo-controlled clinical trials.

## Complement system inhibition may be useful for promoting brain repair after ischemic stroke

Stroke is one of the significant causes of mortality and morbidity worldwide. The only treatment available for patients stricken with ischemic stroke is systemic thrombolysis with tissue plasminogen activator (tPA) (94, 95). Due to an increased risk of bleeding when administered beyond 4.5 hours after stroke, only a smaller percentage (1-2%) of stroke patients can benefit from tPA. About a third of stroke patients die, and among survivors of stroke, 90% of patients suffer permanent deficits (96, 97). Therefore, comprehending the various mechanisms underlying pathogenesis after stroke has immense value for developing an apt therapy that promotes brain repair. Following an ischemic stroke attack, restoration of blood flow occurs in the infarct region, either by endogenous thrombolytic system activation or induced via exogenous thrombolytic therapy [31]. However, cerebral blood flow reperfusion initiates a cascade of pathophysiological events that aggravates brain tissue damage and leads to more severe brain dysfunction and cognitive impairments [98].

While the precise molecular mechanisms underlying cerebral ischemia or reperfusion injury are unknown, studies imply that complement activation plays a significant role in pathophysiology after ischemic stroke [99]. Ma colleagues reviewed the role of complement components in the pathophysiology of ischemic stroke and discussed potential therapeutic strategies for targeting the complement system after ischemic stroke [31]. The complement system has a protective role, as it eradicates pathogens and debris and is an essential element of innate immunity. In many pathological situations, the self-protective action of the complement system could trigger immune, inflammatory, and degenerative responses. The authors highlight that the evidence supporting the involvement of the complement system in the pathogenesis of ischemic brain injury is strong. Notably, the complement system elicits an inflammatory cascade and contributes to tissue injury. Such activity is also evident from observations of diminished pro-inflammatory mechanisms and brain injury when specific components of the complement system were inhibited [31, 100]. Many inhibitors have been tested in animal models, which include C1-INH (inhibitor of both classical and lectin pathways), CVF (inhibitor of C3), sCR1 (C3 convertase inhibitor), IVIg (IgG extracted from the plasma of healthy donors), C3aR antagonist, anti C5 monoclonal antibodies, and a C5aR1 antagonist [31].

However, authors caution that while many of these inhibitors showed potential for modulating or reducing complement system activation after ischemic stroke in animal models, the availability of complement inhibitors for clinical translation remains limited. Another caveat is that several components of the complement system such as C1q, C3, and C3aR are also involved in synaptic plasticity and neurogenesis in both standard and stroke conditions [101]. Such activity raises a question of whether inhibition of the systemic complement system would also block some of the beneficial effects mediated through synaptic plasticity and neurogenesis [31]. Authors propose additional studies focused on examining the effects of complement inhibition at different time-points (e.g., subacute and chronic phases) after ischemic stroke, which may unravel positive and negative effects of complement inhibition on regeneration and remodeling, apart from its beneficial effect for curtailing neuroinflammation.

## Conclusions

The various findings discussed in this commentary have implications for comprehending pathogenesis as well as developing therapeutic strategies for systemic and brain aging, ALS, AD, and stroke. The notable insights related to systemic aging include how: (i) overweight leads to muscle loss and alters mitochondrial metabolism; (ii) an intricate network of many inflammatory mediators converge to cause chronic inflammation; (iii) modulation of miR-34a may promote healthy heart and brain; (iv) enhanced expression of SIRT3 may reduce cardiac and pulmonary fibrosis; and (v) intensive statin therapy can reduce the degree of stenosis in AICAS patients. Additional articles on systemic aging suggested that age-related loss of brain white matter is linked to chronic systemic inflammation, hypertension is a result of reduced miR-181b-5p transforming VSMCs by increasing HMGB1, cataract formation occurs via deficiency of Yes-associated protein, several miRs and lncRNAs have potential as biomarkers and therapeutic targets for the CAD, higher meat consumption can cause sleep problems, and poorer glycemic control is associated with depression. Many other studies provided insights on brain aging, ALS, AD, and stroke. First, individuals with higher education and living in a rural environment seem to have a higher risk of developing ALS. Second, collagen XIX alpha 1 appears to be a useful prognostic biomarker and a therapeutic target in ALS. Third, it seems that extracellular Aβ-42 initiates intraneuronal neurofibrillary tangle accumulation in familial AD. Fourth, TOMs may be useful for slowing down the progression of AD, if additional validation studies in animal models rule out toxicity, infections and long-term efficacy. Fifth, inhibition of the complement system appears to be a useful approach for promoting brain repair after stroke, if the potential side effects of complement inhibitors are ruled out with additional long-term studies in animal models.
